# Evaluation of a mobile safety center's impact on pediatric home safety knowledge and device use

**DOI:** 10.1186/s40621-020-00254-1

**Published:** 2020-06-12

**Authors:** Leah Furman, Stephen Strotmeyer, Christine Vitale, Barbara A. Gaines

**Affiliations:** 1 University of Pittsburgh School of Medicine, Pittsburgh, PA USA; 2 Department of Pediatric General and Thoracic Surgery, UPMC Children’s Hospital of Pittsburgh, Pittsburgh, PA USA

**Keywords:** Gun lock, Home safety, Mobile safety center, Safety products, Smoke detector

## Abstract

**Background:**

A Mobile Safety Center (MSC) is designed to remove financial accessibility barriers to home safety by providing education and safety devices within local communities. The objective of this study was to evaluate the impact of an MSC on pediatric home safety knowledge and device use.

**Methods:**

We conducted a prospective home safety interventional study. Parents and grandparents with children at home were recruited at community events attended by the MSC. Participants completed a pre-test survey assessing demographics and current home safety knowledge, practices, and device use. Participants then attended the MSC’s short home safety educational program. Afterwards, participants completed a knowledge reassessment post-test and were offered free safety devices: a smoke detector, a gun lock, and a childproofing kit comprising outlet covers, doorknob covers, and cabinet latches. We administered two follow-up surveys four weeks and six months after visiting the MSC. Descriptive statistics, Friedman tests, Wilcoxon Sum-Rank tests, and Pearson Chi-Square were used to assess respondent demographic characteristics and changes in home safety knowledge, practices, and device use.

**Results:**

We recruited 50 participants, of whom 29 (58%) completed follow-up 1, 30 (60%) completed follow-up 2, and 26 (52%) completed both. Participants who completed both follow-ups increased total correct answers to safety knowledge questions between the pre-test and post-test (*p* = 0.005), pre-test and follow-up 1 (*p* = 0.003), and pre-test and follow-up 2 (*p* = 0.012) with no significant changes between the post-test, follow-up 1, and follow-up 2. Of the respondents who reported accepting safety products, outlet covers were used most frequently, followed by the smoke detector, doorknob covers, cabinet latches, and the gun lock.

**Conclusions:**

The MSC may be an effective means of increasing home safety among families with children, as participation in the MSC’s home safety educational program significantly increased home safety knowledge and spurred home safety device use. Implementation of MSCs could potentially reduce childhood injury rates within communities through promotion of home safety.

## Background

Unintentional injury is the leading cause of death for children older than 1 year and was responsible for nearly 6 million non-fatal pediatric injuries in the United States in 2017 (Web-based Injury Statistics Query and Reporting System (WISQARS), 2019). Home injuries, many of which are preventable, account for between 40 and 65% of all unintentional child injuries (Morrison et al., 1999; Pitt et al., 1994; Hu et al., 1993). Barriers to child injury prevention include a general lack of safety information; poor timing of safety education, particularly at the time of a child’s birth; inadequate or expensive safety devices; and parental inability to anticipate injuries (Smithson et al., 2011; Ablewhite et al., 2015; Ingram et al., 2012). Facilitators include provision of simple, durable safety devices; ability of parents to predict injury risk; community involvement; face-to-face education; and using methods tailored to each family’s needs (Smithson et al., 2011; Ablewhite et al., 2015; Ingram et al., 2012; Kendrick et al., 2013). Many of these barriers and facilitators could be addressed by a safety center with a comprehensive injury prevention program. Indeed, the SAFE Home Project, or “SAFE trial,” found personalized safety counseling and access to reduced-cost devices at a safety center were effective at improving home safety practices (Gielen et al., 2002).

In a follow-up study to the SAFE trial, barriers to visitation at a safety center were explored; for example, the studied center’s limited schedule provided no hours outside of a typical workday (McDonald et al., 2003). This issue is particularly salient for families of lower socioeconomic status (SES), who may have inflexible job scheduling and limited access to transportation. The accessibility barrier is further compounded by the inverse relationship between SES and rates of unintentional pediatric injury (Bishai et al., 2002; Faelker et al., 2000; Pomerantz et al., 2001; Durkin et al., 1994; Yuma-Guerrero et al., 2018; Cubbin & Smith, 2002; Fallat et al., 2006; Osborne et al., 2016; Gielen et al., 2012). To address the heightened risk of injury among low-SES families, some safety centers have created mobile safety centers (MSCs). MSCs potentially eliminate financial and transportation barriers and bolster community involvement by attending community events.

There are limited studies evaluating MSCs. These studies suggest MSCs are viewed positively by visitors and may increase safety knowledge and some behaviors (Gielen et al., 2009; Bulzacchelli et al., 2009). However, one study did not conduct follow-up and subsequently lacked long-term impact data (Gielen et al., 2009). Another study conducted follow-up but did not utilize the “mobile” aspect of the center. Instead, the MSC used during the latter study was periodically parked at a health center where participants had a preexisting relationship with healthcare providers (Bulzacchelli et al., 2009).

Regardless of mobile capability, the goal of any safety center should be injury rate reduction. The HOME injury study found installation of multiple safety items designed to reduce home injury hazards led to a 70% reduction in rate of modifiable medically attended injury. The study authors concluded there is significant value in the provision and use of safety products (Phelan et al., 2011). Yet recent data from a pediatric clinic suggest even freely-distributed safety items are not always used at home (Habermehl et al., 2019). Identifying “most used” items and focusing on the distribution of these higher-yield items in the future could increase the odds that families use the items they receive from a safety center or MSC.

In this study, we investigated whether an MSC could increase home safety knowledge and device use. We endeavored to mimic the settings that our MSC would attend in the future by recruiting participants through community events. Additionally, we examined which items were most frequently chosen to better tailor the items distributed at future events.

## Methods

To evaluate the MSC, we conducted a prospective home safety interventional study approved by the University of Pittsburgh Institutional Review Board. The MSC attended six community events during a two-month period in 2018. The events were open to the public and held in low-income neighborhoods, except for one event, which was held for employees of a local company. One study team member distributed recruitment flyers to all event attendees. Parents or guardians (18 years or older) were eligible to participate in the study if they had children (less than 18 years old) living in their home. Some participants were grandparents caring for grandchildren.

**Fig. 1:**
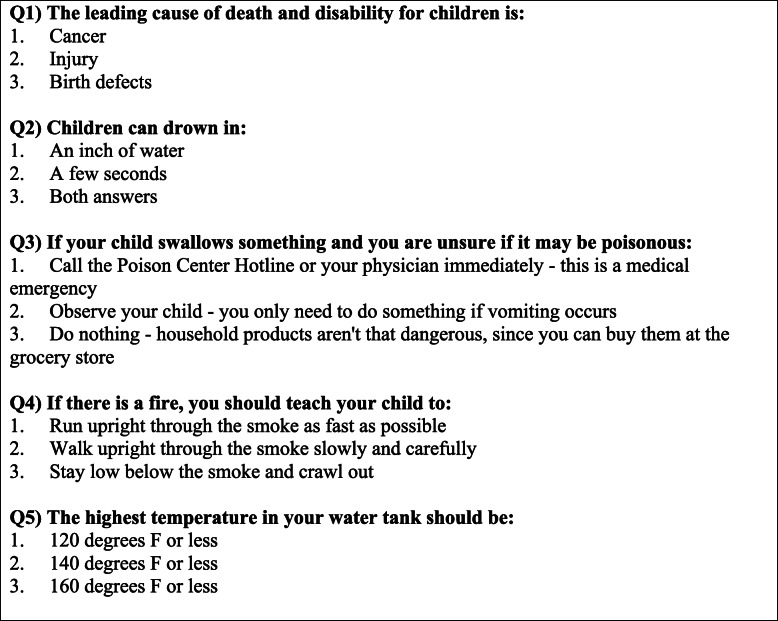
Five home safety knowledge questions participants answered during the pre-test, post-test, follow-up 1, and follow-up 2. Answer key: Question 1, Answer 2. Question 2, Answer 3. Question 3, Answer 1. Question 4, Answer 3. Question 5, Answer 1

After obtaining written informed consent, participants completed pre-tests comprising 12 questions assessing participant demographics, five questions assessing home safety knowledge (Fig. 1), and 17 questions assessing home safety behaviors. Most of these pre-test survey questions were modified from a similar survey previously used by the Safety Center at the University of Pittsburgh’s Medical Center (UPMC) Children’s Hospital of Pittsburgh as an assessment tool for conducting home safety evaluations prior to individualized education. This initial survey took approximately 10–15 min to complete and was scored at a Flesh-Kincaid reading grade level of 4.6 (Microsoft® Word® Version 16.29.1). If a participant was unable to read, a study team member read each survey question and accompanying answer choices verbatim and entered each participant’s answer accordingly. We also requested email addresses and phone numbers and asked that participants designate follow-up contact preferences (email, voice call, or text message).

Participants then attended the comprehensive safety program offered by the MSC. This program followed a standardized curriculum identical to the Safety Center at UPMC Children’s Hospital of Pittsburgh and was taught by trained Safety Center staff members. Participants were taught in multiple small groups or one-on-one depending on event attendance, and education lasted approximately 20 min total. Topics included: safety in the kitchen, living/dining areas, and bathroom, and fire, firearm, and fall safety. All knowledge questions tested were covered during the program.

After completing the program, participants answered a five-question post-test identical to the five knowledge questions asked during the pre-test (Fig. 1). Afterwards, participants were given $5 gift cards and offered free safety items: a smoke detector, a childproofing kit containing outlet covers, doorknob covers, and cabinet latches, and a gun lock. Participants could choose all three, a combination, or none. Pre-test and post-test surveys were conducted on provided tablet computers using Qualtrics® (Provo, Utah) or on paper copies of the printed Qualtrics® survey, depending on participant preference.

We contacted participants to complete follow-up surveys 4 weeks after attending the program (follow-up 1). Follow-up 1 comprised five knowledge questions identical to the ones on the pre-test and post-test, 17 questions assessing home safety behaviors and risk factors identical to those on pre-test, and between one and 11 questions assessing use of items distributed, depending on how many items were taken. For text and email follow-up, we distributed links to a Qualtrics® survey for participants to complete. When calling, the study team member read each question and all answer choices from an identical Qualtrics® survey verbatim over the phone to the participant and entered the selections. Contact occurred at fixed intervals for a total duration of 21 days (if call preferred) or 22 days (if text/email preferred). We spaced calling contact slightly differently than texting/email to maximize variation in days of the week, as active text/email links could be answered at any convenient time. Regardless of modality, we contacted each participant at five unique timepoints, and began by only using the preferred modality. If a participant gave other modalities of contact, we simultaneously used these in conjunction with the preferred modality after two unsuccessful attempts. We accepted responses for 2 months after the first contact.

A week prior to follow-up 2, we notified participants that completion of this follow-up would automatically qualify them for a random chance to receive a $25 gift card. Notifications were made via call or email. Then, 6 months post-MSC visit, we contacted participants to complete follow-up 2, with questions identical to follow-up 1. Contact occurred at fixed intervals as described above. Due to an initially non-working text link, we added an extra contact to all participants 1 week following the previous end point of contact. The winner of the gift card was notified 1 week after the study was closed for responses.

We examined demographics using range, median, and frequency percentiles. Participant answers to knowledge questions were scored as either 1 (correct) or 0 (incorrect) and totaled for all 5 knowledge questions. Unanswered knowledge questions were scored as a 0. We calculated mean and standard deviation for total scores and used Friedman and Wilcoxon sum-rank (WSR) testing to identify significant differences between both total and individual pairs of scores. We analyzed item usage with Friedman and WSR testing and used Pearson Chi-Square analysis to assess mode effect. Concordance percentages were calculated by adding the number of participants who reported using an item during both follow-ups to the number who reported not using the item on both follow-ups and dividing by the total participants who reported receiving the item on both follow-ups. For all statistics, a *p*-value less than 0.05 was considered significant. Results were analyzed using IBM® SPSS® Statistics Version 25 (Mission Hills, CA).

## Results

### Respondent demographics

We enrolled 50 participants. The study population was primarily female (90%) and was ethnically/racially similar to the population of Pittsburgh (Table 1). All participants had at least an 8th grade education; 80% completed high school or equivalent, and 24% had a bachelor’s degree or higher. Ages ranged from 23 to 71 years, with a median of 37 years. Most participants were married or in a domestic partnership (60%) and employed (56%). Median annual income, $25,000–$34,999, was lower than the $44,092 median annual income in Pittsburgh (United States Census Bureau QuickFacts Pittsburgh city, Pennsylvania, 2018). Contact information was provided by 48 (96%) participants, with 29 contactable participants completing follow-up 1 (60.4%), 30 completing follow-up 2 (62.5%), and 26 completing both (54.2%) (“complete follow-up group”).

**Table 1:** Demographic breakdown of gender, race and education of study population compared to the population of Pittsburgh^a^

Demographic Information		Study Population, ***N*** = 50 n (%)	Pittsburgh Population Census Estimate (%)(United States Census Bureau QuickFacts Pittsburgh city, Pennsylvania, 2018)
**Gender**	**Female**	45 (90)	(51.1)
	**Male**	5 (10)	(48.9)
**Race**	**Asian/Pacific Islander**	5 (10)	(5.6)
	**Black or African American**	10 (20)	(23.6)
	**Caucasian**	29 (58)	(64.7)
	**Hispanic or Latino**	3 (6)	(2.9)
	**Multiracial**	2 (4)	(3.4)
	**Native American or American Indian**	1 (2)	(0.2)
**Education**	**High School Graduate, Diploma or Equivalent or Higher**	40 (80)	(92.1)
	**Bachelor’s Degree or Higher**	12 (24)	(41.9)

^a^Pittsburgh population estimates based on data provided by the United States Census Bureau. Of note, educational statistics for the population of Pittsburgh are for adults age 25 years and older, as compared to 23 and older for our population

### Knowledge

**Fig. 2:**
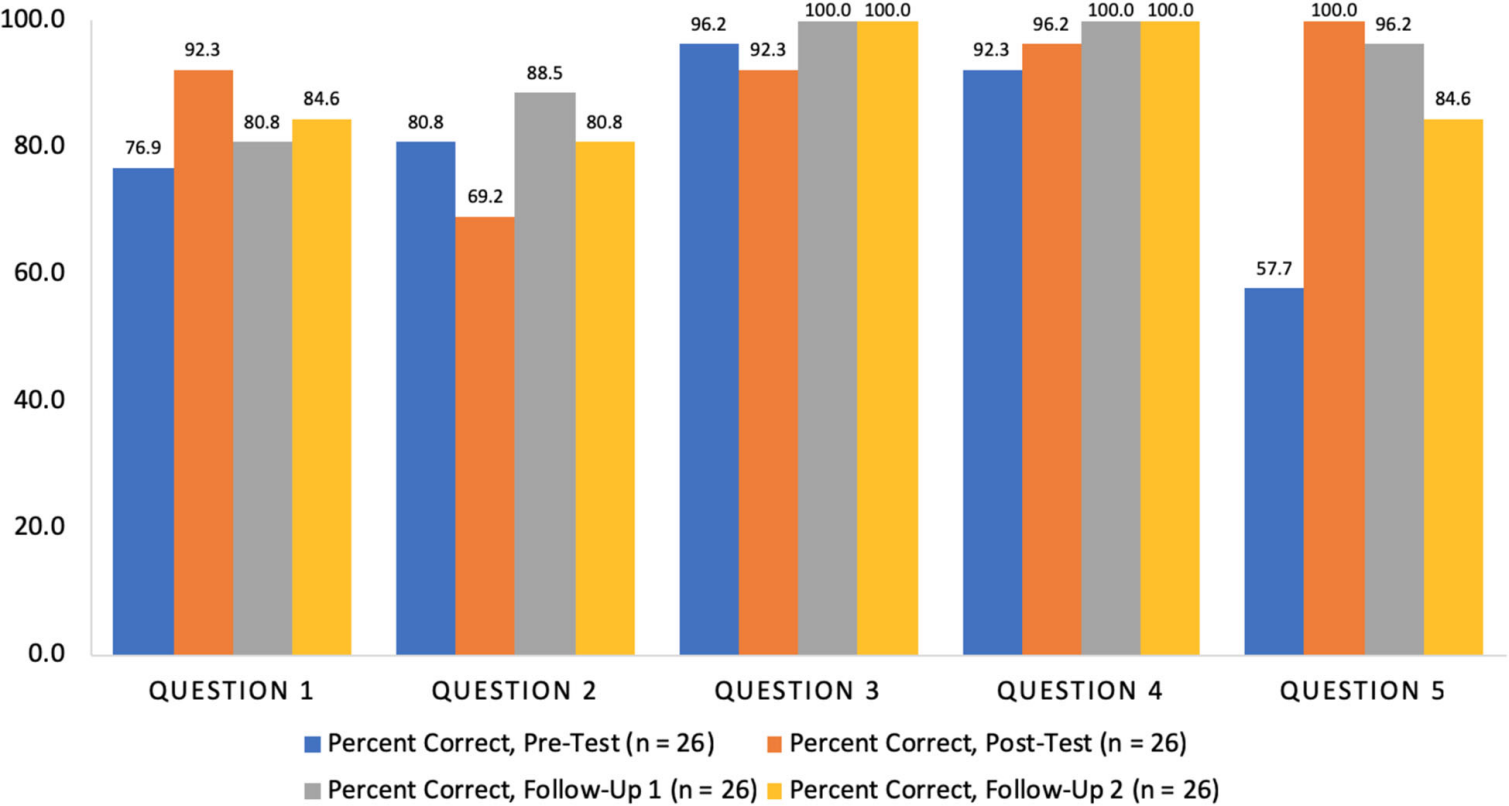
Percentage of the complete follow-up group (n = 26) who answered each knowledge question correctly on the pre-test, post-test, follow-up 1, and follow-up 2

When comparing changes in scores across tests for the complete follow-up group, scores on Questions 1 through 4 did not demonstrate significant differences, but there were significant differences for Question 5 (*p* < 0.001). Question 5 (“The highest temperature in your water tank should be:”) was the most challenging for participants, as it was answered correctly by only 66% (33/50) of total participants on the pre-test, and 57.7% (15/26) of the complete follow-up group (Fig. 2). WSR testing for Question 5 revealed significant score improvement between the pre-test and the post-test (*p* = 0.001), follow-up 1 (*p* = 0.002), and follow-up 2 (*p* = 0.02), although the second follow-up score was significantly lower than the post-test score (*p* = 0.05).

**Fig. 3:**
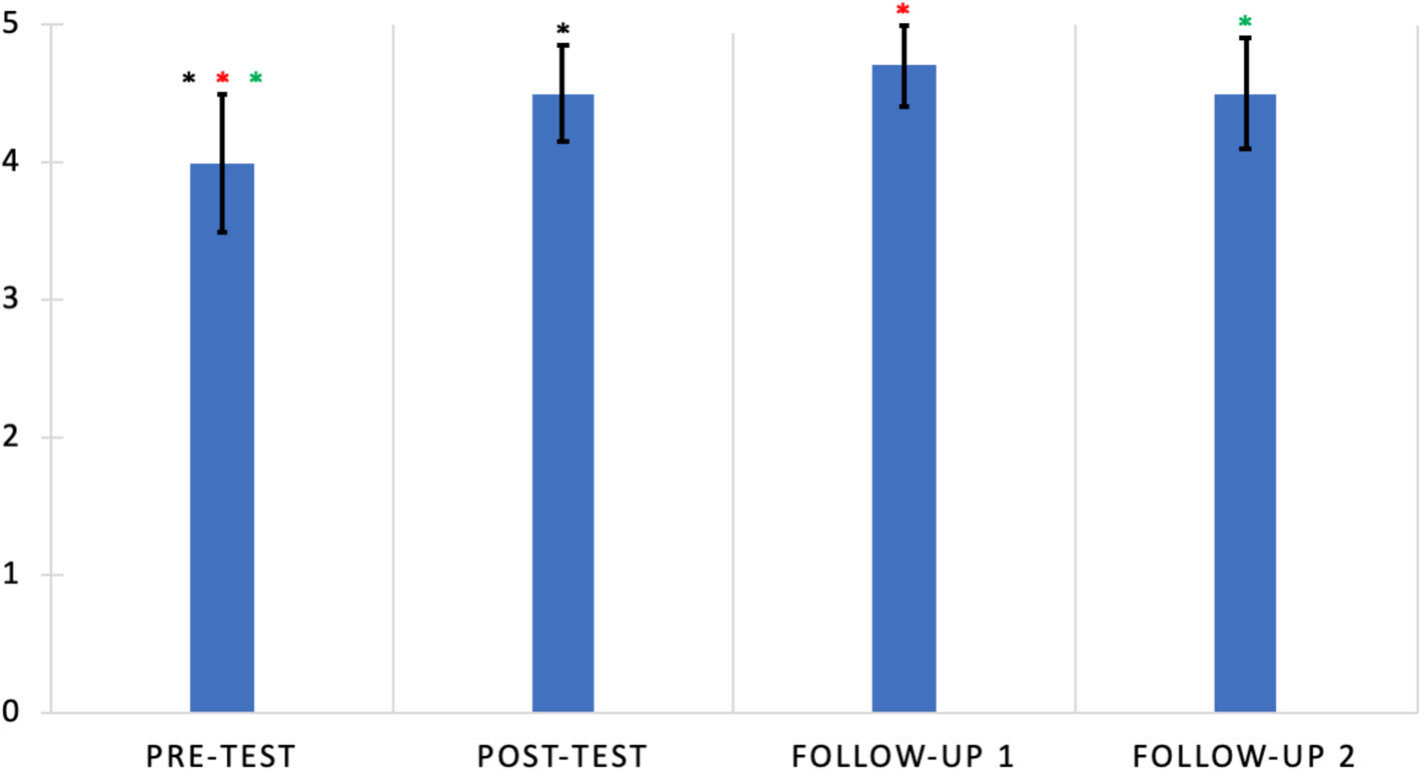
Mean total scores for the complete follow-up group (*n* = 26) were significantly increased from pre-test to post-test, and these significant increases were maintained at both follow-ups. Wilcoxon sum-rank testing of each pairing individually revealed significant differences in scores between the pre-test and each other score (pre-test vs. post-test, p = 0.005; pre-test vs. follow-up 1, p = 0.003; pre-test vs. follow-up 2, p = 0.012), but no other significant differences between pairs. Error bars represent standard deviation. Identically colored pairs of stars indicate significant differences between pairs

Friedman testing of total scores from the complete follow-up group showed a significant difference between mean total scores, with mean scores of 3.9 ± 0.6, 4.5 ± 0.4, 4.6 ± 0.4, and 4.4 ± 0.5 for the pre-test, post-test, follow-up 1 and follow-up 2, respectively (Fig. 3). WSR testing of individual pairs of mean total scores for this group showed all other mean total scores were significantly increased from the pre-test mean total score (pre-test vs. post-test, *p* = 0.005; pre-test vs. follow-up 1, *p* = 0.003; pre-test vs. follow-up 2, *p* = 0.01). However, mean total scores after the pre-test were not significantly different from each other.

### Device use

Of follow-up 1 respondents, 27/29 (93%) accepted at least one item, while 29/30 (97%) of follow-up 2 respondents did so. The childproofing kit was the most common choice. Outlet covers were most often reported to be in-use at both follow-ups (Table 2). Friedman testing showed usage of the three items in the childproofing kit was significantly different for both follow-ups (*p* = 0.002 for follow-up 1 and *p* = 0.006 for follow-up 2). WSR testing demonstrated outlet covers were used significantly more frequently than either doorknob covers or cabinet latches for follow-up 1. For follow-up 2, outlet covers were only used significantly more frequently than cabinet latches (Table 3).

**Table 2:** Device reception and use at first and second follow-up

*Item*		*Follow-Up 1 (n = 29)*			*Follow-Up 2 (n = 30)*		
		# Received Item	# Currently Using	% Currently Using	# Received Item	# Currently Using	% Currently Using
*Child-Proofing Kit*	**Doorknob Covers**	26	15	57.7%	25	18	72%
	**Outlet Covers**	26	22	84.6%	25	22	88%
	**Cabinet Latches**	26	12	46.2%	25	12	48%
*Non-Child-Proofing Kit*	**Smoke Detector**	19	16	84.2%	22	19	86.4%
	**Gun Lock**	19	4	21.1%	20	5	25%

Items in the childproofing kit (outlet covers, doorknob covers, cabinet latches) are listed separately than those not in the kit (smoke detector, gun lock) to reflect the usage differential of the individual items

**Table 3:** Differences in childproofing kit item usage during follow-up

	Follow-Up 1			Follow-Up 2		
Wilcoxon Sum-Rank Pairs	Outlet Covers + Doorknob Covers	Outlet Covers + Cabinet Latches	Doorknob Covers + Cabinet Latches	Outlet Covers + Doorknob Covers	Outlet Covers + Cabinet Latches	Doorknob Covers + Cabinet Latches
**Significance**	p = 0.008*	p = 0.002*	*p* = 0.32	*p* = 0.21	p = 0.002*	*p* = 0.06

Friedman testing of childproofing kit item usage for both follow-ups revealed significant differences (*p* = 0.002 for follow-up 1, and p = 0.006 for follow-up 2). Significant *p*-values are indicated in italics with a star

We noted some discrepancies between follow-ups regarding item acceptance. For childproofing kits, 21 participants consistently reported receiving one, while three participants responded differently between follow-ups (concordance rate = 87.5%). Gun locks had a similar concordance rate of 85%, while smoke detector reports varied more, with a concordance rate of 56.5%. Among participants who consistently reported receiving an item, reports of whether they were using the item also sometimes differed between follow-ups (Table 4). Electronic (text and email) vs. person-to-person (calling) mode effect was examined with Pearson Chi-Square analysis and only significantly impacted doorknob cover usage during follow-up 1 (*p* = 0.04), with participants more likely to answer affirmatively when asked over the phone. None of the other items met statistical significance.

**Table 4:** Item usage reports for respondents who reported receiving the item on both follow-ups

	Follow-up 1 Yes, Follow-Up 2 Yes	Follow-up 1 Yes, Follow-Up 2 No	Follow-up 1 No, Follow-Up 2 Yes	Follow-up 1 No, Follow-Up 2 No	Follow-Ups in Concordance, Percentage
**Doorknob Covers** **(*n*** **= 21)**	9	2	5	5	66.7%
**Outlet Covers** **(n = 21)**	16	1	3	1	81.0%
**Cabinet Latches** **(n = 21)**	5	5	5	6	52.4%
**Smoke Detector** **(*n*** **= 13)**	11	0	1	1	92.3%
**Gun Lock** **(*n*** **= 17)**	3	1	1	12	88.2%

Items in the childproofing kit (outlet covers, doorknob covers, cabinet latches) are listed separately from those not in the kit (smoke detector, gun lock)

### Gun safety

During the pre-test, 11 participants reported at least one firearm in their home. For storage at baseline, six participants used a gun safe, one used a gun lock, one used a locked cabinet or drawer, and three used a high place. At follow-up 1, four of the 11 participants who initially reported a firearm at home were lost to follow-up. The remaining seven included one individual who had previously reported owning a firearm then reported they did not own a firearm, and six who continued to report a firearm at home. For storage, five participants reported using a gun safe, and one continued to store it in a high place. At follow-up 2, the same six participants reported a firearm at home, and one person added a gun lock in addition to the gun safe. Interestingly, three participants consistently denied having a firearm at home, but reported taking and using a gun lock.

## Discussion

Our study examined the use of an MSC as a tool for increasing home safety knowledge and device use, particularly among low-SES populations. Through community event attendance, we successfully recruited low-SES participants, increased home safety knowledge long-term, and distributed free safety products. We also found reported product usage varied considerably between items.

### Respondent demographics

The MSC chose community events as recruitment sites to best reflect the future population that would be primarily served by the MSC. While most of these events served a low-income population, we included one event held at a local company to recruit a small number of middle- and higher-income participants. These events were chosen as our recruitment sites to best reflect the future population that would be served by the MSC: primarily low-income populations with some attendance from middle- and higher-income populations.

Overall, participants in our study had a lower median income, were less likely to have completed high school, and had fewer advanced degrees than the general population of Pittsburgh (United States Census Bureau QuickFacts Pittsburgh city, Pennsylvania, 2018), indicating that the MSC was successful in reaching lower-SES communities. This is particularly crucial given the association between low SES and higher rates of pediatric injury (Bishai et al., 2002; Faelker et al., 2000; Pomerantz et al., 2001; Durkin et al., 1994; Yuma-Guerrero et al., 2018; Cubbin & Smith, 2002; Fallat et al., 2006; Osborne et al., 2016; Gielen et al., 2012). We suggest our success in contacting lower-SES populations was largely due to our ability to bring the MSC to community events, thus leveraging the mobile capability of the MSC to primarily focus on the most at-risk population.

### Knowledge

The five questions used to assess knowledge gains examined a range of content presented in conjunction with various aspects of home safety. Only Question 5, “The highest temperature in your water tank should be:,” was answered significantly differently across time points, with pre-test scores significantly lower than any subsequent scores. Notably, Question 5 was the question answered incorrectly most often prior to the educational program, suggesting participants learned the answer during the program. However, the percentage of participants answering Question 5 correctly dropped significantly between the post-test and follow-up 2, perhaps indicating some knowledge loss. Despite this, both mean follow-up scores were still significantly higher than the mean pre-test baseline, suggesting that the MSC increases knowledge long-term.

Totaling knowledge scores magnified smaller changes seen for Questions 1 through 4. Altogether, participants increased their total score from the pre-test to the post-test and sustained this increase at both follow-ups. No significant decrease in knowledge occurred for total knowledge scores, reinforcing the indication that MSC education increased safety knowledge long-term. While our significance seemed to be primarily driven by Question 5, Question 5 was possibly the most representative of true “knowledge gains,” because, as noted above, it was the question answered correctly least often on the pre-test. Our results echo Bulzacchelli et al.’s finding that visiting a stationary, health center-associated MSC was effective at increasing safety knowledge (Bulzacchelli et al., 2009). Furthermore, our gains were made in participants attending community events, a situation that is better reflective of the effectiveness of the MSC.

### Device use

The discrepancies in reported item acceptance highlight a limitation of the study design: we allowed participants to choose which items to take without independently recording their choices. Our design likely reduced item redundancy and waste, as families who already owned an item could choose not to take another. Our methods may also have reduced the stigma some families felt by taking all items, particularly regarding gun locks. However, this impacted our ability to know with certainty which items each participant took.

We did not ask participants why they did or did not use an item. However, some possible reasons include: ease or difficulty of installation, perceived importance of safety product, and whether they understood the purpose of the item. We found of the three items in the childproofing kit, outlet covers were reportedly used significantly more frequently than cabinet latches at both follow-ups. This difference could reflect that these items are at the extremes of difficulty for installation, as the outlet covers can just be plugged in as-is while the cabinet latches required home hardware to install.

We found a significant mode effect when comparing usage reported electronically or over-the-phone only for doorknob cover usage on follow-up 1, with more participants affirming usage over the phone. This could be respondent bias secondary to a desire to please the study team by asserting use when interacting more directly. However, doorknob covers were also the first item that participants were asked about, suggesting participants who may have felt pressured to report usage did not feel the need to do so more than once.

### Gun safety

We assured all participants of confidentiality during the consent process and provided standard gun safety education without asking about gun ownership. Despite this, we considered whether the stigma and political polarization around gun ownership would impact the truthfulness of our results. Interestingly, the data did contain notable discrepancies in reports about ownership and storage across survey timepoints that the authors felt warranted further discussion.

During the pretest, one participant reported a firearm at home, but then denied this during follow-ups. Intriguingly, several participants denied having a firearm in the home, but reported using the gun lock that we had given to them. As mentioned earlier, discrepancies existed between follow-up 1 and follow-up 2 regarding reports of whether a participant took an item and used it. It is possible participants picked an answer unintentionally, or circumstances changed between follow-ups. It is also possible participants felt uncomfortable admitting to owning a firearm, but still used the gun lock provided by the MSC. This brings us to a key conclusion: blanket provision of education and tools promoting gun safety, without assessing ownership status, could be effective at changing behaviors.

### Limitations

Our study had some notable limitations. As noted above, we did not independently record which safety items that each participant took, so we do not know with certainty the safety items obtained by each study participant. As with most survey-based studies, our responses were subject to participant recall and social desirability bias. We also recruited participants at community events with the same population that we intend to reach with the MSC in the future. In doing so we conducted convenience sampling, which may have been impacted by selection bias. Also, our sample size was small, and we lost over 40% of participants to follow-up, suggesting our follow-up results may have been further impacted by selection bias. Additionally, as shown in the mode effects analysis, our results may have been influenced by responder bias, as we relied on self-report. Of note, studies designed to explore the validity of self-report for home safety practices have been generally good to mixed, with some reporting accuracy as high as 100% for certain practices and others demonstrating over-reporting 17–24% of the time. However, it is worth noting these studies relied on home observation checks to validate parental report, which in itself may introduce bias (Lee et al., 2012; Hatfield et al., 2006; Yorkston et al., 2005). Finally, while our study did show some significant increases in safety knowledge, further work would be needed to determine whether this difference is clinically meaningful.

## Conclusions

We conclude the MSC may increase home safety among families by expanding safety knowledge and spurring the use of some freely distributed devices. Certain items, such as outlet covers and smoke detectors, were used by families significantly more frequently, and it may be prudent to prioritize the distribution of these items in the future. Importantly, stationing the MSC at community events is effective at reaching low-SES families, a population particularly vulnerable to unintentional child injuries. Employment of MSCs could potentially reduce community-wide childhood injury rates through comprehensive home safety interventions.

## Data Availability

The datasets used and/or analyzed during the current study are available from the corresponding author on reasonable request.

## Abbreviations

MSC: Mobile safety center
SES: Socioeconomic status
UPMC: University of Pittsburgh Medical Center
WSR: Wilcoxon sum-rank
